# Microfluidic encapsulation of enzymes and steroids within solid lipid nanoparticles

**DOI:** 10.1007/s13346-023-01398-5

**Published:** 2023-07-28

**Authors:** Edward Weaver, Federica Sommonte, Andrew Hooker, Nunzio Denora, Shahid Uddin, Dimitrios A. Lamprou

**Affiliations:** 1 School of Pharmacy, Queen’s University Belfast, 97 Lisburn Road, Belfast, BT9 7BL UK; 2 Department of Pharmacy-Pharmaceutical Sciences, University of Bari “Aldo Moro”, 4 Orabona St., Bari, 70125 Italy; 3 Immunocore Ltd., 92 Park Dr, Milton, Abingdon, OX14 4RY UK

**Keywords:** Solid lipid nanoparticles, Microfluidics, Biologics, Enzymes, Testosterone, Nanoformulation

## Abstract

The production of solid lipid nanoparticles (SLNs) is challenging, especially when considering the incorporation of biologics. A novel in-house method of microfluidic production of biologic-encapsulated SLNs is proposed, using a variety of base materials for formulation to help overcome the barriers presented during manufacture and administration. Trypsin is used as a model drug for hydrophilic encapsulation whilst testosterone is employed as a positive non-biologic lipophilic control active pharmaceutical ingredient. Particle sizes obtained ranged from 160 to 320 nm, and a lead formulation has been identified from the combinations assayed, allowing for high encapsulation efficiencies (47–90%, respectively) of both the large hydrophilic and the small hydrophobic active pharmaceutical ingredients (APIs). Drug release profiles were analysed in vitro to provide useful insight into sustained kinetics, providing data towards future in vivo studies, which displayed a slow prolonged release for testosterone and a quicker burst release for trypsin. The study represents a large leap forward in the field of SLN production, especially in the field of difficult-to-encapsulate molecules, and the technique also benefits from being more environmentally sustainable due to the use of microfluidics.

**Graphical Abstract:**

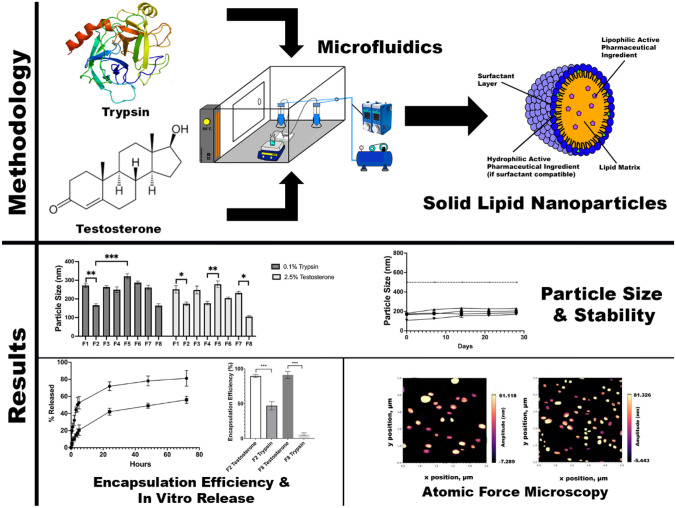

**Supplementary Information:**

The online version contains supplementary material available at 10.1007/s13346-023-01398-5.

## Introduction

To enable biologics for a more proficient and patient-accepted delivery, it is essential that they be protected from inhospitable internal conditions caused by the vast array of proteases and pH climates that exist post-administration. Biologics are, by nature, large and complex molecules with various subregions of differing electrochemical properties [1], which can often present as being a challenge to encapsulate within nanosystems. Biologic molecules are manufactured or derived from biological (living) sources; this means that they encompass a wide range of drug products, from insulins to mRNAs. The latter mentioned group here has gained drastic interest since its widespread usage as an mRNA vaccine to protect against COVID-19 [2]. Biologics are potent but expensive materials to produce, meaning their formulation route should be optimised, to ensure the capacity for a viable medicine to be mass produced for marketing. The interest in oral delivery too is an area of growing interest due to increased patient compliance and the non-invasiveness of administration. To achieve both a pharmaceutically viable and patient compliant formulation, one proposed method is nanoformulation: particularly within solid lipid nanoparticles (SLNs), to act as both a physical and chemical barrier for the active pharmaceutical ingredient (API) post-administration.

**Fig. 1:**
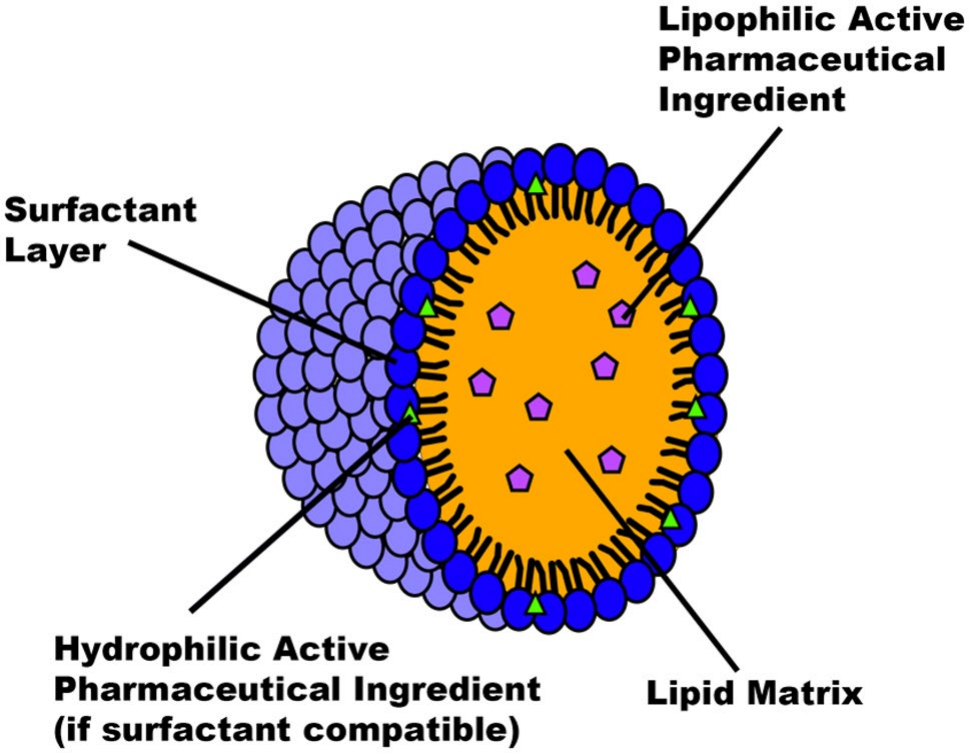
General structure of an SLN capable of encapsulating an API

SLNs have been shown as a potent means for achieving targeted, sustained release for various APIs, including chemotherapeutics [3], genetic material [4], and anti-rheumatics [5]. Since their first conception in 1996 [6], the technology has developed to allow for a wider scope of APIs to be internalised, as well as improving particle characteristics such as stability, targeting, and size [4, 7]. The structure of an SLN can allow for the encapsulation of both hydrophilic and hydrophobic molecules, due to the hydrophobic core and hydrophilic external layer (Fig. 1). The solid core is the primary structural difference to that of a liposome (LP), which possesses an aqueous vacuole. The lipid core consists usually of a physiological lipid which remains solid at both room and body temperatures, such as waxes, sterols, and glycerides [8]. The core is often responsible for containing the API payload, especially for hydrophobic APIs and it has been proven in that the contents of the solid core affects the release profile of an API from an SLN [9]; this is partly owing to the interactions between the API and the core. The choice of API will dramatically affect the chosen SLN components; for example, as concluded by Botto et al., a cationic lipid core and surfactant are essential to optimise the formulation of mRNA and DNA within an SLN [10]. The external surfactant layer is mainly responsible for promoting the stability and targeted delivery of the nanoparticle but can also act as the containment area for hydrophilic APIs [11].

Within the area of lipid-based nanoparticles, e.g. SLNs, LPs, niosomes (NIOs), and exosomes (EXOs), each type of formulation has their own unique properties, as shall be summarised in Table 1. For comparative purposes, NIOs are being included in this category of lipid-based therapies, despite unmodified NIOs not containing raw lipids.

**Table 1:** A summary of the different characteristics of all the common lipid-based nanoformulations

Nanoformulation type	Formulation materials	Capable of encapsulating	Advantages	Disadvantages
SLNs	• Waxes • Sterols • Surfactants [8]	• Hydrophobic and hydrophilic [12]	• Highly modifiable • High biocompatibility, including non-toxic degradation [13]	• Require a cooling process for solidification • API leakage during storage • Complications caused by crystallisation [14]
LPs	• Phospholipids • Cholesterol [15]	• Hydrophobic and hydrophilic [16]	• Highly modifiable • Used for theranostic purposes [17] • Simple synthesis	• Low skin permeability [18] • Infrequently possess low mechanical strength [19]
NIOs	• Non-ionic surfactants • Cholesterol [20]	• Hydrophobic and hydrophilic [21, 22]	• Biocompatible and non-immunogenic [22] • Improve drug permeation through the skin • Less stringent storage requirements compared to LPs	• Time-consuming to create • API leakage [22]
EXOs	• Lipids • Proteins • Glycoconjugates [23]	• Hydrophobic and hydrophilic • Genetic material [24]	• Used for theranostic purposes [25]	• Complex and Expensive to artificially manufacture [26]

There are however limitations for the exponential growth of SLN applications, some of which are briefly mentioned in Table 1. A large limitation is a concise and reproducible method for their production. Historically, bulk production methods such as homogenisation (e.g. cold, hot, shear, ultrasonic) or microemulsification have been used for the production of SLNs [4], although these methods frequently suffer from pitfalls including unpredictable particle characteristics [27], not mentioning their environmentally detrimental use of large volumes of solvents [28]. It has therefore proposed that a more novel usage of microfluidics (MFs) be employed to combat these issues of formulation. Previous attempts of engaging MFs are limited but have shown promising initial results. To the authors’ knowledge, the only previous applications of MF for the production of biologic-encapsulated APIs were performed by Anderluzzi and Perrie [29] and Sommonte et al. [30] who used a different formulation method as to that which is proposed in this manuscript. The method proposed in the mentioned works represents a leap in the manufacturing of SLNs; however, questions surrounding the scalability of the method could arise, leaving the need for a more suitable scale-up procedure.

MFs as a formulation platform for drug delivery have increased in popularity over the last decade, with recent research highlighting the capacity to produce a wide range of nanoformulations, including lipid-based formulations [31] and polymeric [32] and metal colloids [33]. The capacity to control various parameters within the technology, including the total flow rate (TFR), flow rate ratio (FRR), device design, and mixing angles within submicron channels, has lead to a large amount of interest in the technology. A commonly praised attribute belonging to MFs is the time efficiency of the process, as formulations are able to be produced in a continuous fashion within minutes of preparation time. This is greatly reduced from more traditional formulation methods, such as film hydration, which is a batch process that can occupy hours of preparation followed by the subsequent need for post-processing. Formulation processes that rely upon the phenomenon of ‘self-assembly’, such as liposomes, SLNs, and lipid nanocapsules, are ideal candidates for MFs. The range of APIs that have been incorporated into nano-/microformulations is also diverse, ranging from chemotherapeutics [34] to gene therapy [35]. The recent COVID-19 pandemic has also lead to an increase in MF use, with a focus upon vaccine-based RNA delivery, using MFs as a formulation platform.

The synergy of MFs with other emerging technologies, such as additive manufacturing, has also allowed for the complex and efficient design of MF devices for unique formulation purposes. This is critical, as the impact on final formulation properties that occurs due to the actual device and the channel geometries is highly cited in literature [36]. Formulation properties that have been seen to have been controlled via MFs are particle size, morphology, and encapsulation efficiency, amongst others [37].

SLNs have often incurred limitations in their loading capacity, due to a compact waxy core and storage crystallisation [38]. As such, it is essential that a method of production be devised that will facilitate the highest initial loading to account for any API lost during the cooling phase. MFs have already seen the encapsulation efficiency (EE) of many nanoformulations increase, including LPs [39, 40] and NIOs [41]. Despite the limited research, data could suggest that the system could increase the EE for SLNs too [29]. Research utilising MFs for encapsulation of lysozyme within SLNs increased the EE% from 53% using a benchtop preparation method to 70%, highlighting the potential benefits of the this emerging new method [30]. One of the aims of this research is to attempt the encapsulation of various APIs using MFs to determine whether this trend is followed. The choice of materials for SLN fabrication will also be varied to allow for the comparison between compositions. Cetyl palmitate (CP) in conjunction with Pluronic F68 (P68) is a common combination of materials for SLN production and is one that has already been attempted with MFs by Arduino et al. [42]. This combination, alongside others previously attempted using bulk methods, will be the basis of SLN materials used to define whether MFs are a more efficacious method for SLN preparation compared to traditional methods. The main important factor to control during the MF manufacture of SLNs is the temperature at which they are produced. As mentioned, the solid core should remain solid both during storage and post-administration (≈ 37 °C); however, during the MF manufacturing process, all components must either be liquid or remain in solution. Achieving these high-temperature conditions is far easier using the traditional methods (e.g. homogenisation) [43], which is likely the reason that has been studied in more depth. Equalling this feat is more of a challenge using a MF setup, which is why the research performed in this study will explore a novel method by ensuring the whole environment is sufficiently temperature controlled.

MFs itself allow the precise control of various parameters during manufacture within a confined volumetric environment, including the TFR, FRR, mixing angles, and duration. Both the TFR and FRR have been demonstrated to have a statistically significant effect upon the particle size of cetyl palmitate SLNs [44]; hence, it is hypothesised that these parameters will affect the size of other combinations too. The reproducibility of formulations from MF systems [45] is one of the major factors contributing towards the surge of interest in MFs as a manufacturing method, which, as mentioned, is in dire need for the formulation of SLNs. This research is aimed at further exploring this area of drug formulation, with the intent of proving that a MF approach could be at the forefront of SLN production in general and for biologic SLNs specifically.

In this study, two APIs have been chosen to analyse for their compatibility for SLN encapsulation using MFs: trypsin (TRP) and testosterone (TES). These were chosen to allow the comparison between their differing sizes and hydrophilicities upon the effect of final particle characteristics. It should be noted that testosterone is regulated as a drug molecule rather than a biologic by the FDA; however, due to its naturally occurring nature and previous synthesis route, the two molecules act as a reasonable comparison. TES has previously been encapsulated within SLNs multiple times [46, 47], however, to the knowledge of the authors, never via a MF method. TES has been chosen additionally to reinforce the propensity of MFs as a manufacturing method for SLNs, to investigate whether the quality of nanoformulation is comparable to previously attempted research with ‘traditional methods’, e.g. homogenisation. TRP was chosen, as the use of biologics within SLNs is extremely limited, especially a hydrophilic API such as TRP; hence, an attempt to display the possibility of encapsulating a large hydrophobic molecule within the nanosystem has been undertaken. A combination of SLN materials was used for the nanoproduction, including tripalmitin (Tri-P), soybean lecithin (LEC), and Tween 80 (T80), as well as the previously mentioned CP/P68.

## Materials and methods

### Materials

**Fig. 2:**
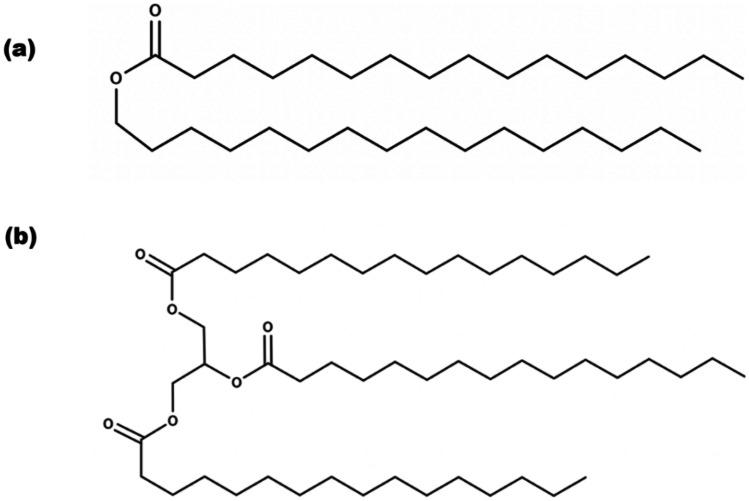
Chemical structures displayed for the core materials for the production SLNs: **a** CP and **b** Tri-P

**Fig. 3:**
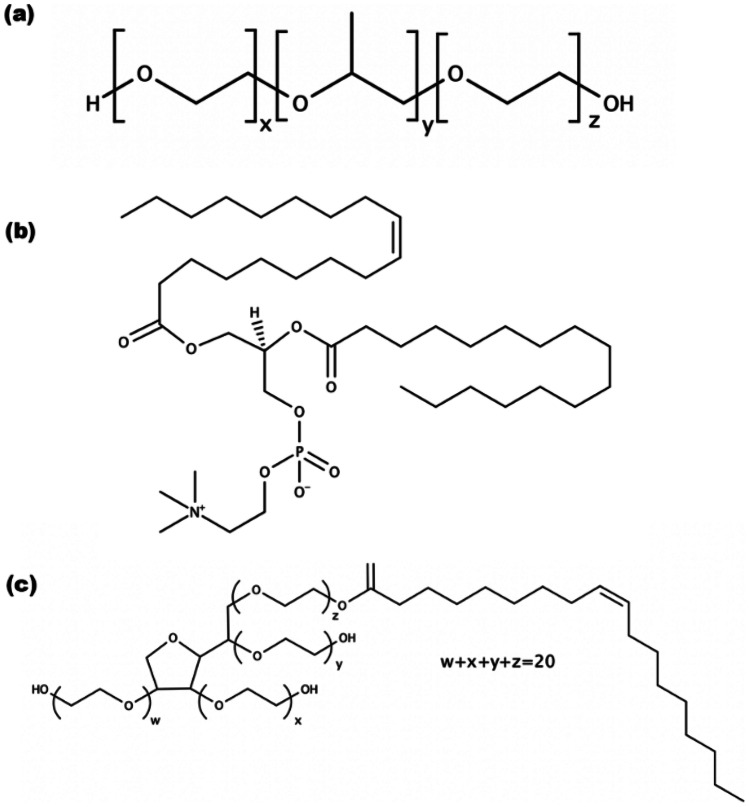
Chemical structures for the surfactants used to produce SLNs: **a** P68, **b** lecithin from soybean, and **c** Tween 80

CP, Tri-P, LEC, P68, TRP, and TES (≥ 98%); ethanol (≥ 99.8%); methanol (≥ 99.9%); Tween 80; and phosphate-buffered saline (PBS (pH 7.4)) were purchased from Sigma-Aldrich. Chemical structures of SLN material can be seen in Figs. 2 and 3.

### Microfluidic preparation of solid lipid nanoparticles

**Fig. 4:**
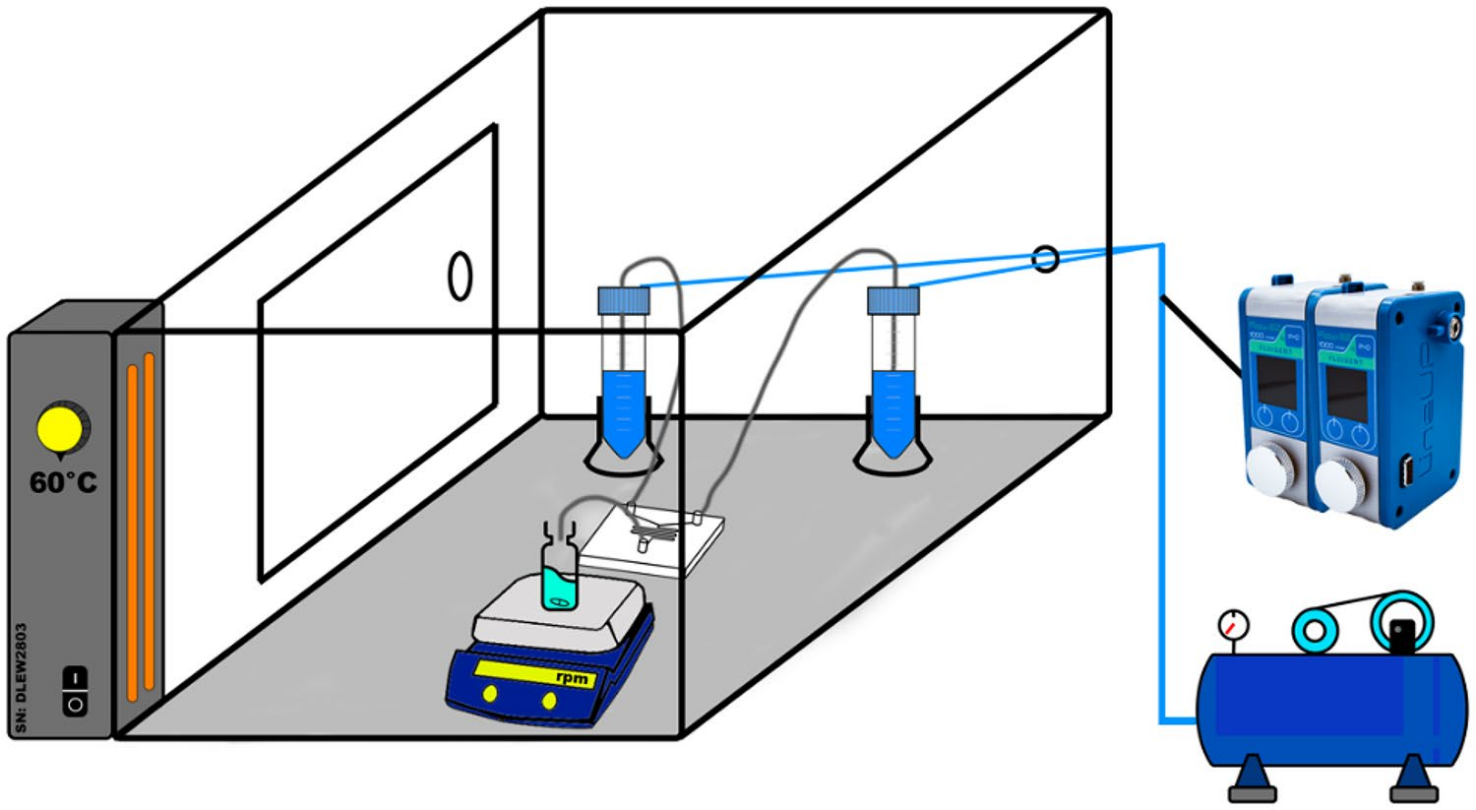
Microfluidic in-house setup for the production of SLNs at a constantly elevated temperature

The Fluigent (Paris, France) Lineup Flow EZ™ Microfluidic system was used to synthesise various formulations of SLNs. To ensure complete dissolution of the materials used throughout, the temperature of the microfluidic environment was maintained at 60 °C as displayed in Fig. 4.

Various concentrations and combinations of SLNs were produced and have been named F1–F8. All materials were dissolved at 60 °C at a mass that was equivalent to their final concentration in the MF chamber (reservoir) solutions. The precise combination of materials used can be seen in Table 2.

**Table 2:** Combinations and concentrations of SLN-preparatory materials used during synthesis

	Material used (% in reservoir solution w/v)				
Formulation	Tri-P	CP	LEC	Tween 80	P68
F1		1			2
F2		0.5			1
F3		1	2	2	
F4		0.5	1	1	
F5	1				2
F6	0.5				1
F7	1		2	2	
F8	0.5		1	1	

For API-encapsulated SLNs, either TRP or TES was used using a 0.1% or 2.5% w/v final concentration respectively. TRP was dissolved in the aqueous phase, whilst TES was dissolved in the organic phase. A FRR of 5:1 (aqueous to organic) was determined to be optimal after empty SLN production (Online Resource 1), using a TFR of 4 ml min^−1^. Samples were collected and excess ethanol was evaporated using constant vortex stirring for 45 min. SLN formulations were placed then at 5 °C for 24 h. The MF device used for manufacturing was the Y-shaped inlet chip produced by Precision NanoSystems (Vancouver, Canada) for the Nanoassemblr Benchtop, with etched herringbone channels.

### Physicochemical characterisation

#### Dynamic light scattering and zeta potential

The NanoBrook Omni particle sizer (Brookhaven Instruments, Holtsville, NY, USA) was used to analyse particle size and polydispersity index (PDI). Each measurement was performed in triplicate, using 20 μl of nanoparticle (NP) suspension diluted in 1900 μl of PBS. Zeta (ζ) potential was measured also by NanoBrook Omni using the SREL solvent-resistant electrode probe. A total sample size of 1920 μl was used for each assay, after dilution. Samples were prepared in triplicate, then measured in triplicate, meaning a total of 9 samples per formulation were assayed.

#### Stability studies

Particle size, PDI, and ζ-potential readings were measured over the course of a 28-day period, recording values every 7 days, beginning on day zero of preparation. Samples were kept in constant conditions in triplicate, at two temperature values of 5 °C and 37 °C. The 5 °C storage represents viable storage conditions of a formulation to investigate the change in physical particle characteristics, whereas the 37 °C mimics the formulations’ physical behaviour at in vivo temperatures.

#### Atomic force microscopy

AFM was completed using a TT-2 AFM (AFMworkshop, USA) to investigate particle morphology and to assist in corroborating the DLS results. SLN formulations were diluted to the same concentration as used for DLS. The diluted samples were placed on a freshly cleaved mica surface (1.5 cm × 1.5 cm; G250-2 Mica sheets 1″ × 1″ × 0.006″; Agar Scientific Ltd., Essex, UK) and left to dry for 1 h, before rinsing with DI water to remove excess nanoparticles. Ohm-cm Antimony doped Si probes were used for analysis (frequency range 311–344 kHz), and scans were performed at a scan rate of 0.6 Hz and a resolution of 512 × 512 pixels.

#### Encapsulation efficiency (EE) and drug release

Centrifugation, dynamic dialysis, and high-performance liquid chromatography (HPLC) were employed to carry out EE and drug release. SLN formulations were initially centrifuged for 30 min at 14,800 rpm. Supernatant was removed and kept for analysis of EE. The surface of the remaining SLN was further washed to remove any remaining unencapsulated API and kept for analysis of EE. EE% was calculated as detailed in Eq. 1.

1 $$\mathrm{\%EE}= \frac{\text{Total mass of API added }\left(\text{mg}\right)-\text{Mass of unencapsulated API }(\text{mg})}{\text{Total mass of API added }(\text{mg})}\times 100$$

The remaining pellet post-centrifugation was resuspended and redispersed using mechanical mixing and placed into dialysis tubing (SpectraPOR^®^, 50 kDa, Fisher Scientific, Milan, Italy). For TRP-loaded SLN formulations, 37 °C PBS was used as the release medium, whereas for TES-loaded SLN formulations, 37 °C methanol adjusted to pH 7.4 was used. Measurements were taken from the external medium as 500-μl aliquots and replaced with fresh medium to ensure sink conditions remained constant. Drug release was measured at intervals of 30 min, 1 h, 2 h, 3 h, 4 h, 5 h, 24 h, 48 h, and 72 h. Samples were run in triplicate.

UV-HPLC (Agilent Infinity 1220 LC system (California, USA)) was used to measure respective concentrations of API. A C18 ODS HYPERSIL column (250 × 4.6 mm, particle size 5 μm) from Thermo Scientific (USA) was used. The method for each API is as follows.

For TRP, a mobile phase system of the gradient system consisted of water/trifluoroacetic acid 0.1% (v/v) (A) and acetonitrile/trifluoroacetic acid 0.1% (v/v) (B) was used. The elution gradient followed was 100:0 0–20 min, 50:50 20–25 min, and 0:100 25–35 min (A:B respectively). Each sample was measured over a total run time of 35 min with a column temperature of 45 °C. Flow rate remained constant, at 1 ml min^−1^. UV absorbance was measured at *λ* = 280 nm [15].

For TES, adapted from Butnariu et al. [48], the mobile phase system consisted of HPLC grade methanol at a flow rate of 1 ml min^−1^ for 10 min. UV absorbance was measured at 240 nm.

#### Differential scanning calorimetry (DSC)

DSC was performed using the Netzsch autosampler (Wolverhampton, UK), using standard aluminium pans. Mass of pan contents varied between 5 and 12 mg. For Tri-P, dynamic heating was performed from 0 to 80 °C at a heating rate of 10 K per minute. For all other assayed materials and formulations, a 0–450 °C heating profile was employed at a heating rate of 10 °C. SLN formulations were centrifuged at 14,800 rpm for 30 min, supernatant removed, and air dried for analysis.

#### Fourier transform infrared spectroscopy (FTIR)

Analysis was performed using the Nicolet is-50 FTIR with built-in ATR (Thermo Fisher Scientific, USA) on solid samples. Solid samples were obtained, when necessary, as documented in the ‘Encapsulation efficiency (EE) and drug release’ section. Scans were performed under an inert atmosphere over a wavelength range of 4000–600 cm^−1^, over 64 scans at a resolution of 4 cm^−1^, and an interval of 1 cm^−1^. Background absorption was subtracted from each scan.

### Statistical analysis

All methods mentioned were performed in triplicate where appropriate. Initial data handling, including standard deviation and average calculations, was completed using Microsoft Excel. One-way ANOVA was completed using Prism 9 software using significance levels of *p ≤ 0.05, **p ≤ 0.01, and ***p ≤ 0.001.

## Results and discussion

### Dynamic light scattering (DLS)

**Fig. 5:**
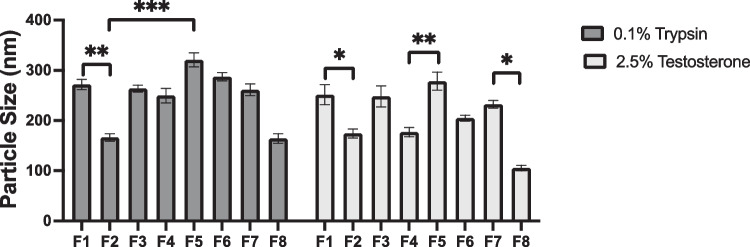
Particle size measurements for formulations F1–F8. All size measurements were taken on day 0 of formulation

DLS results displayed varied results, according to the material combinations used and respective concentrations. Comparing the non-loaded SLNs (Online Resource 1), with the particle size obtained for the API-loaded SLNs (Fig. 5), it is apparent that a similar trend in size is observed with a minimal increase in diameter upon encapsulation. It is apparent that F2 and F8 possess the most favourable particle sizes of the formulations, which is the reason that these were chosen to continue as the model formulations for the duration of the study. The stability studies also included the ‘least-favourable’ formulations, F1 and F5, to act as a negative control. F1 parameters were based upon studies from Sommonte et al. [30], who produced optimal formulations using this combination of concentrations. This was achieved using a self-manufactured MF chip. It was found adversely though in the current study that, when using a commercially available chip and the Fluigent system, halving the required concentrations provided more opportune particle diameter. This factor indicates the importance of considering both the system and the MF environment that is being used for formulation.

The waxy-core materials chosen in this study were aptly selected due to their comparable chemical characteristics. The chemical structure of Tri-P and CP is closely linked, differing by only a carbonyl group and hydrocarbon tail (Fig. 2). When comparing solely the particle size, it is apparent that the choice of surfactant, when coupled with CP, has a low impact on particle size. This is not the case for Tri-P, as using LEC/T80 as the surfactants consistently produces smaller SLNs as compared to P68. The LEC/T80 combination has consistently been used to stabilise the solid cores of SLNs, owing to their non-toxic combination and [49] viable longevity. The additional hydrocarbon tail present in Tri-P, as opposed to CP, is likely the reason for the variation of sizes due to an increased propensity for Van der Waals interactions. The choice of API did not have as significant of an effect on particle size as the choice of materials, as there is no clear difference between particle size between the TRP and the TES. This was unexpected, due to the differing log *p* values of the two APIs causing a relative partitioning position within the SLN. This trend could, however, be caused by the flow restriction of the MF device as the channel diameters are less < 100 μm, negating a significant difference in the self-assembly process that occurs upon formulation, independent upon the choice of API. It appears that the major parameter that affects the size is the interaction between waxy core material and surfactant layer. TRP is relatively small, as far as biologics are concerned, with a molecular weight of approximately 24 kDa [50], which may be suitable for partitioning in the aqueous surfactant layer of P68 at the designed pH of 7.4. Formulations using LEC/T80 surfactants may not offer such a degree of encapsulation potential owing to the hydrophobic nature of the surfactant layer.

### Stability studies

**Fig. 6:**
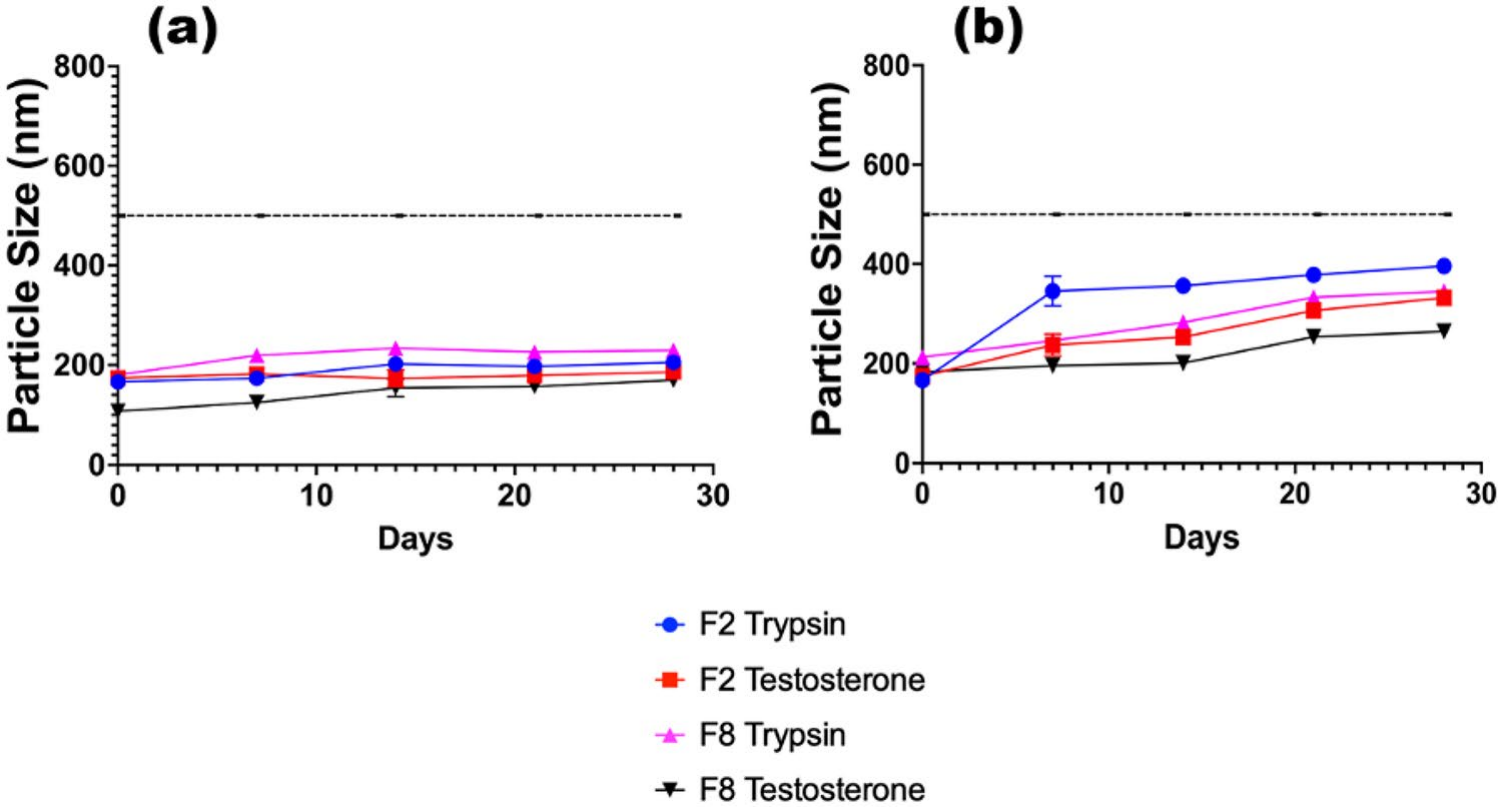
Stability studies taken over a 28-day period for TRP- and TES-loaded F2 and F8 formulations, stored at **a** 5 °C and **b** 37 °C

**Fig. 7:**
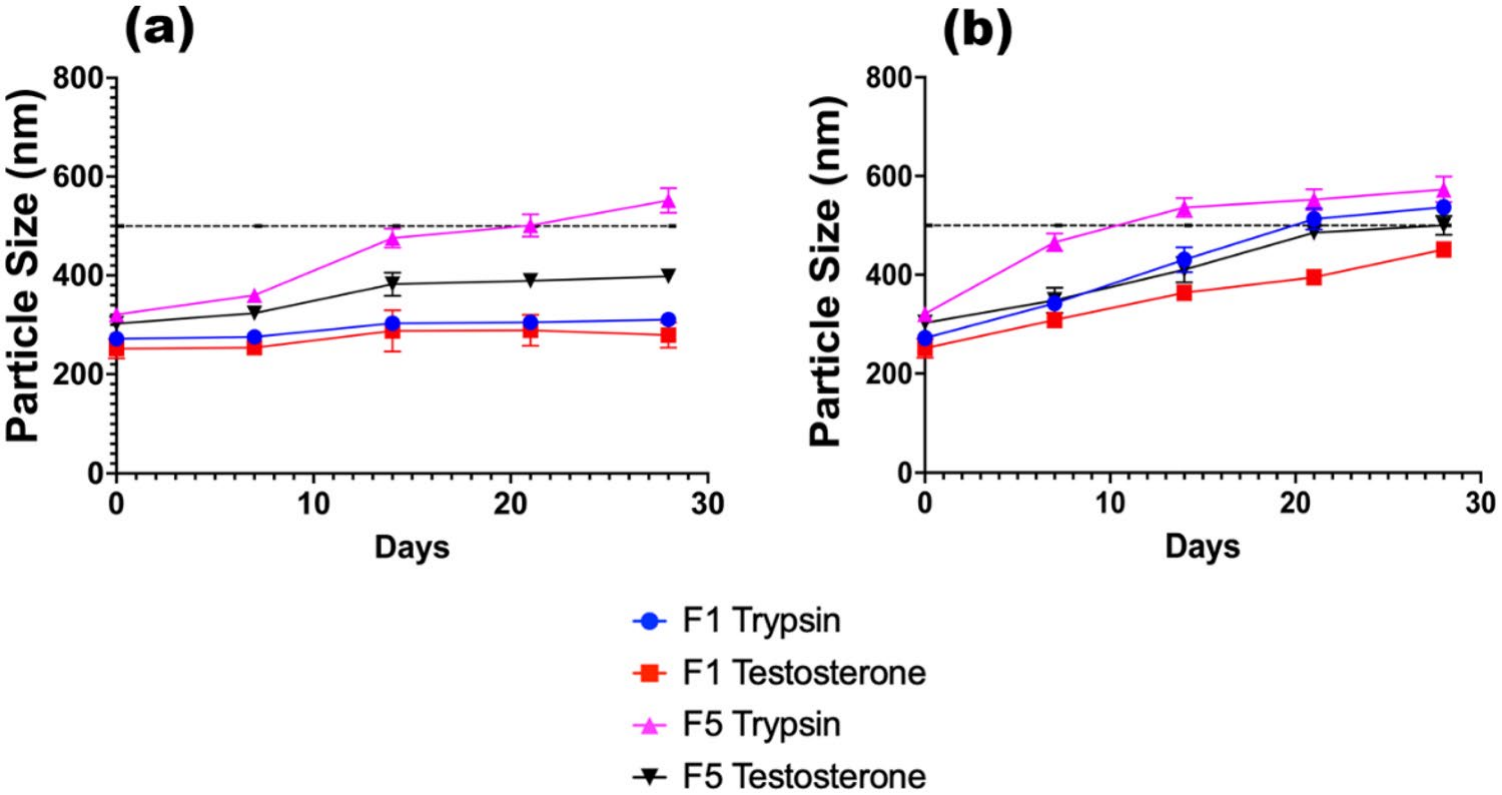
Stability studies taken over a 28-day period for TRP- and TES-loaded F1 and F5 formulations, stored at **a** 5 °C and **b** 37 °C

The physical stability of the formulations is exceptionally promising, at both temperature conditions assayed. SLNs are often cited as having a greater physical stability when examined against LPs, due to their solid properties [51]. There is no clear trend in the data that suggests an optimal core/surfactant combination to choose; however, the overall trend is as expected, that the nanoparticles grow gradually over time, due to aggregation, crystallisation state changes, and surfactant expansion. The stability studies were divided into two strains, depicted in Figs. 6 and 7. This assisted the authors to determine whether the initial formulation size of an SLN had a knock-on effect for its stability, as well as the formulation conditions. Whilst all chosen formulations appeared to function well, it was clear that F2 and F8 had favourable stability characteristics, to provide a consistent prolonged release. The worst performing formulation in terms of stability appeared to be F5 TRP, which coincidentally had the largest initial formulation size. It is theorised that due to the disruption caused by the TRP partitioning within the surfactant layer, the internal stability of the solid core is slightly compromised, causing slight expansion at both temperatures. A complete summary of the PDI and ζ-potential data recorded throughout the experiment is available in Online Resources 5–12. The ζ-potential of the formulations varied slightly throughout the duration of the stability study for TES; however, for TRP formulations, the ζ-potential appeared to become more positive over time. This is likely due to API release during storage. TES is considered a neutral molecule [52] at physiological pH, which provides evidence as to why the ζ-potential of the formulation changed only slightly, whereas trypsin appears to have a slight positive charge due to the increase in positive surface charge. The phenomenon of protein corona formation, as well as liposomal aggregation caused by the increased neutrality of the particles, appears to be the causative factor of increased particle size for TRP formulations.

### Atomic force microscopy (AFM)

**Fig. 8:**
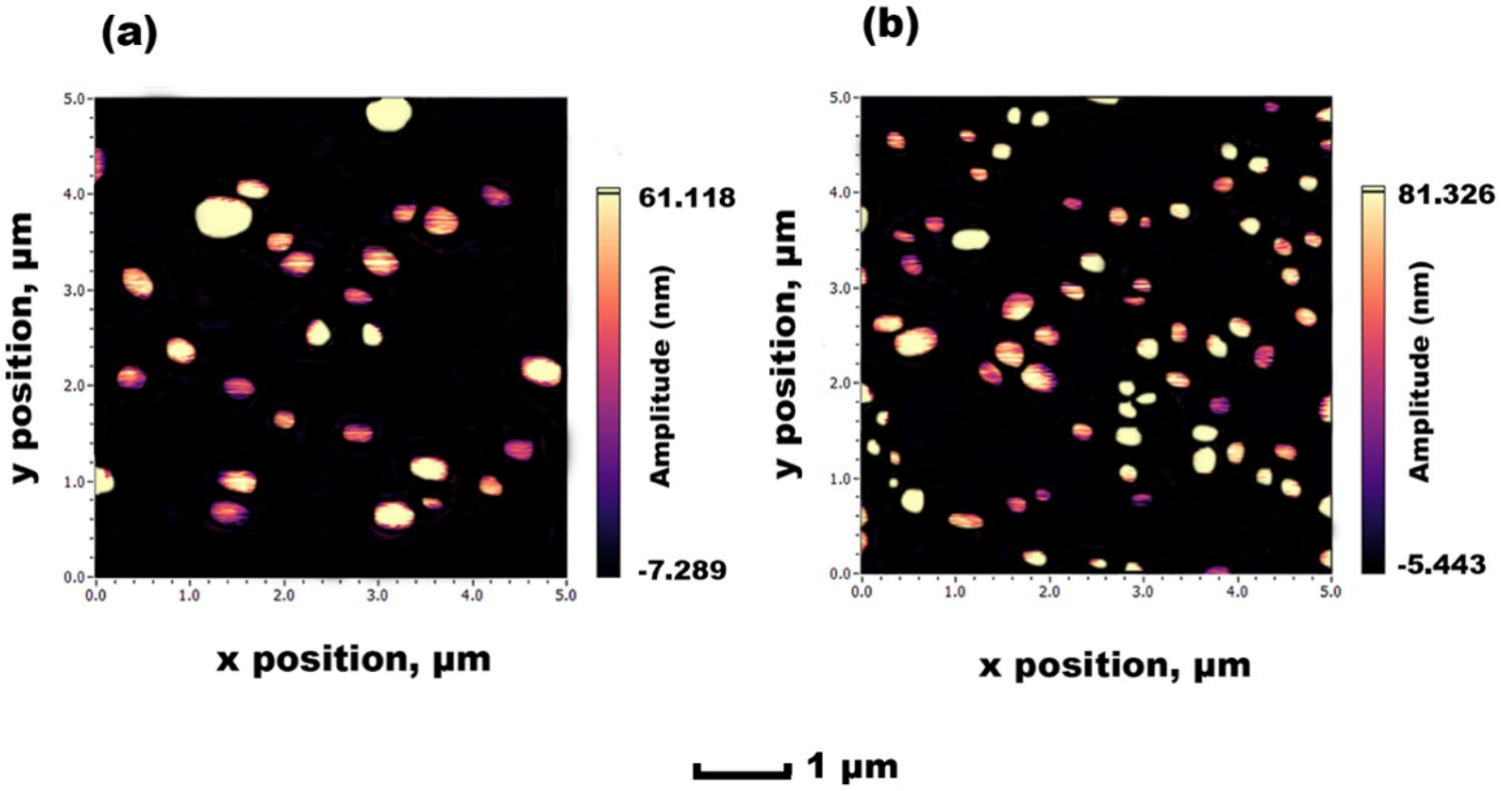
AFM images obtained for F8 SLN encapsulation: **a** TRP and **b** TES

AFM results (Fig. 8) also support the findings of DLS, indicating the size range that had been previously obtained. It is normal for AFM images to be appear slightly enlarged for the diameter due to the drying on substrate stage [53], which is further reinforced by the height profile suggesting a slight flattening. A uniform dispersion of SLNs is visible using AFM, due to the intense wash step detailed in the ‘Atomic force microscopy’ section, to remove any non-adhered nanoparticles from the mica surface. As witnessed, the general physical characteristics displayed for the SLN production appear promising for future development.

### Differential scanning calorimetry (DSC)

**Fig. 9:**
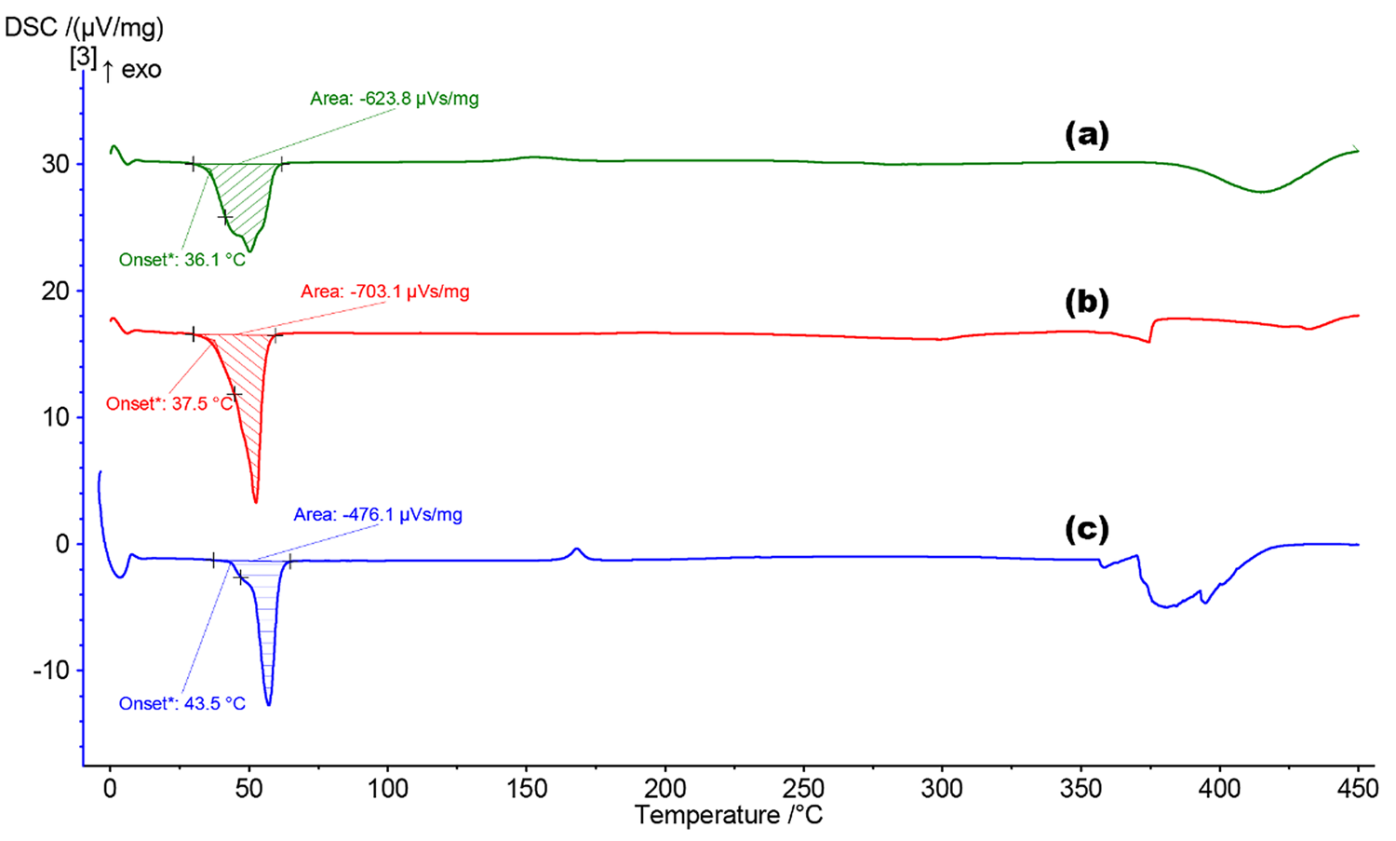
DSC analysis for **a** F2 TRP SLNs, **b** raw CP, and **c** raw P68

DSC was used to analyse whether the thermal stability of the raw materials used was affected during their combination into SLNs. As witnessed with common pharmaceuticals such as EMLA cream, the inter-/intramolecular interactions between solid materials can cause the final formulated product to exist in a different state, e.g. liquid. The determination of the semi-crystalline/amorphous form of the formulation was also important to indicate the relative capacity for encapsulation of APIs. The formulation of CP and P68 into a SLN (Fig. 9) has a noticeable effect upon the thermal stability of the raw materials. The onset of melting temperature of the materials is not affected to a high degree, but the enthalpy for phase transition is lowered. This suggests that upon transitioning the raw materials into a combined form, the structure of the materials tends towards a more readily amorphous structure. The DSC thermogram in Fig. 9c has the suggestive shape of a partially crystalline polymer, due to the elongated slope after onset. The cooling thermogram collected (Online Resource 4) supports this conclusion as a glass transition, *T*_g_, can been seen clearly.

**Fig. 10:**
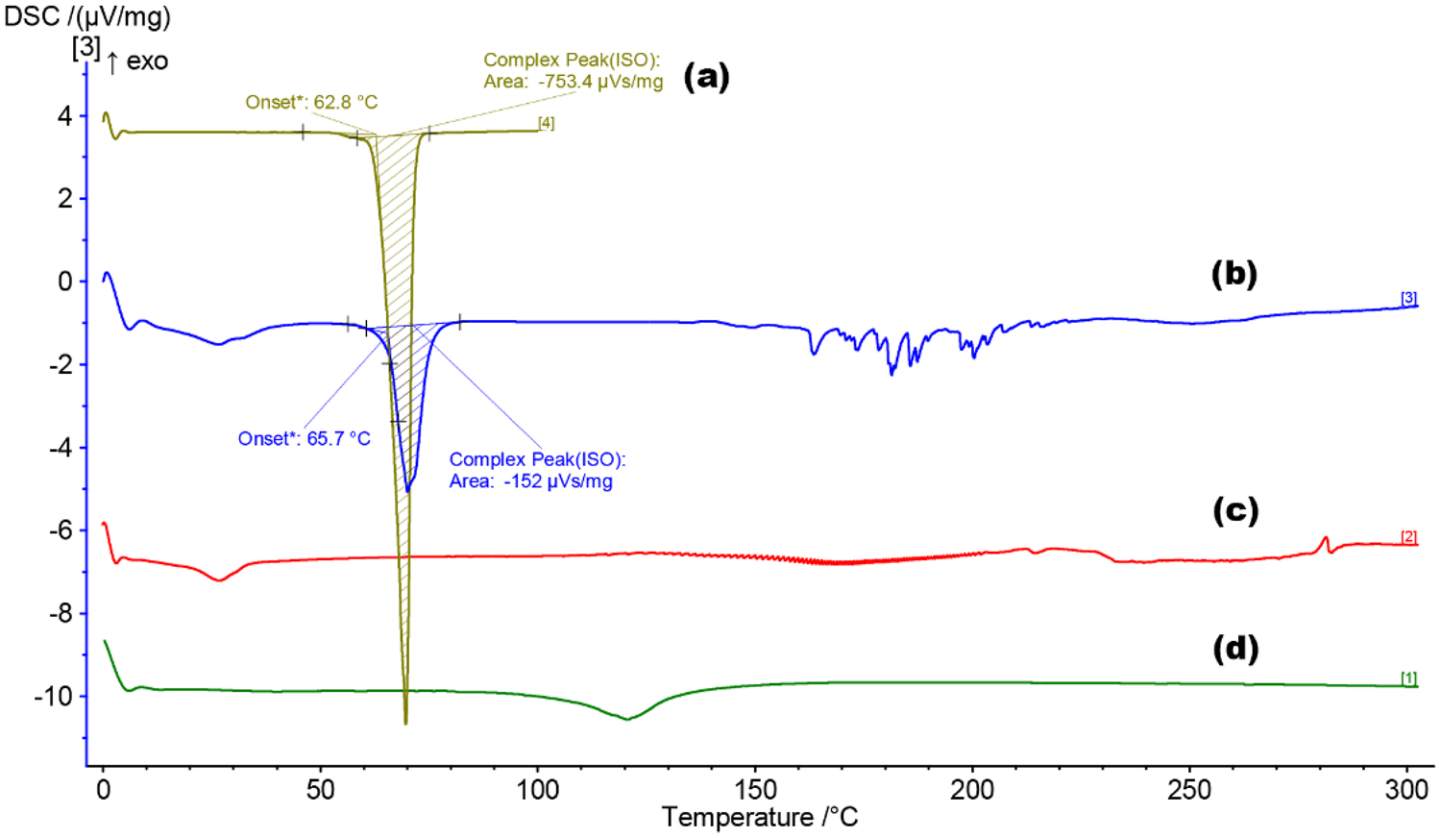
DSC analysis for **a** Tri-P, **b** F8 TRP SLNs, **c** LEC, and **d** T80

The alternative DSC spectra presented (Fig. 10) represents the thermal changes existing for the F8-TRP SLNs. As can be seen in Fig. 10c and d, no major thermal events occurred within the heating range, which was expected for these materials. The encapsulation of TRP within F8 appears to severely reduce the endothermic peak observed around 70 °C. This indicates that the melting transition of the formulation, especially the Tri-P within the formulation, is affected by the encapsulation process.

Not only does the amorphous/crystalline structure have an effect upon API loading; it can also affect the release profile. As noted in multiple studies, amorphous materials allow for a freer movement of API intrastructure [54, 55], meaning that a burst release profile is more likely to occur. The onset of melting for F2 occurs at 36.1 °C, allowing a free movement of API at this point. This is reinforced in the drug release profile (Fig. 14) where a slightly quicker release of both APIs as compared to F8 is achieved.

### Fourier transform infrared spectroscopy (FTIR)

**Fig. 11:**
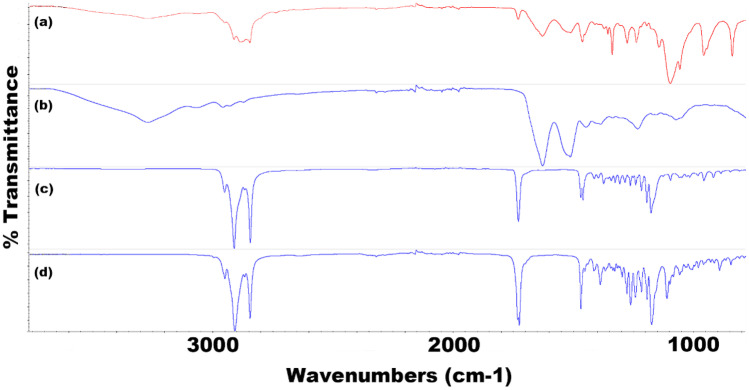
FTIR spectra for all components of F2 TRP depicting **a** F2 TRP-encapsulated SLNs, **b** TRP, **c** CP, and **d** P68

**Fig. 12:**
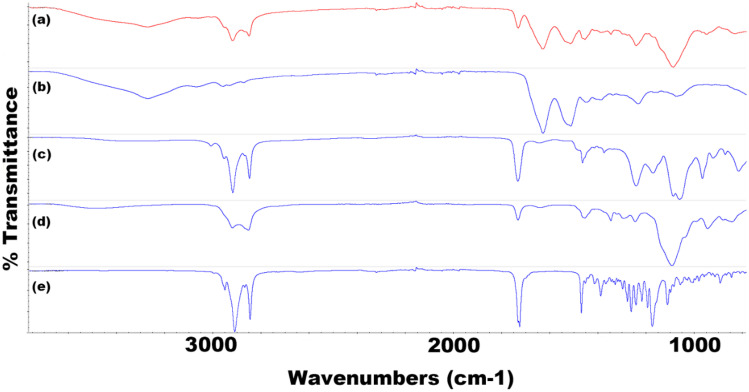
FTIR spectra for all components of F8 TRP depicting **a** F8 TRP-encapsulated SLNs, **b** TRP, **c** LEC, **d** T80, and **e** Tri-P

For TRP-encapsulated SLNs (Figs. 11 and 12), FTIR results clearly display a measurable presence of TRP in the formulation. The broad O–H carboxylic acid stretch at 3260 cm^−1^ is present in the formulation due to the presence of TRP. Further evidence to support the encapsulation in both F2 and F8 can be seen at 1510 cm^−1^ caused by N–O stretching and a C = C monosubstituted stretch within the formulation. Other notable peaks in the formulation include the presence of lecithin within the SLN at 1245 cm^−1^ and 1050 cm^−1^, caused by P = O bonds and P-OR ester groups respectively. Present in most spectra are sharp peaks at 2910 cm^−1^ and 2850 cm^−1^, which are classic of C-H stretching in long alkane chains present within most surfactants and core materials. The spectra in Fig. 12 (F8 TRP) has a notable offset peak at 1090 cm^−1^, due to the C-O primary alcohol absorption stretching.

For TES-encapsulated SLNs (Online Resources 2 and 3), again, the presence of TES within the formulation can be detected using FTIR. The most notable identifying peaks confirming its presence come at 3580 cm^−1^ and 3390 cm^−1^. The medium-sized sharp peaks are present due to the free O–H stretching located on the aromatic penta-carbon ring. The characteristic peaks are present in both the raw material and the SLN formulation confirming the API’s presence.

### Encapsulation efficiency and drug release

**Fig. 13:**
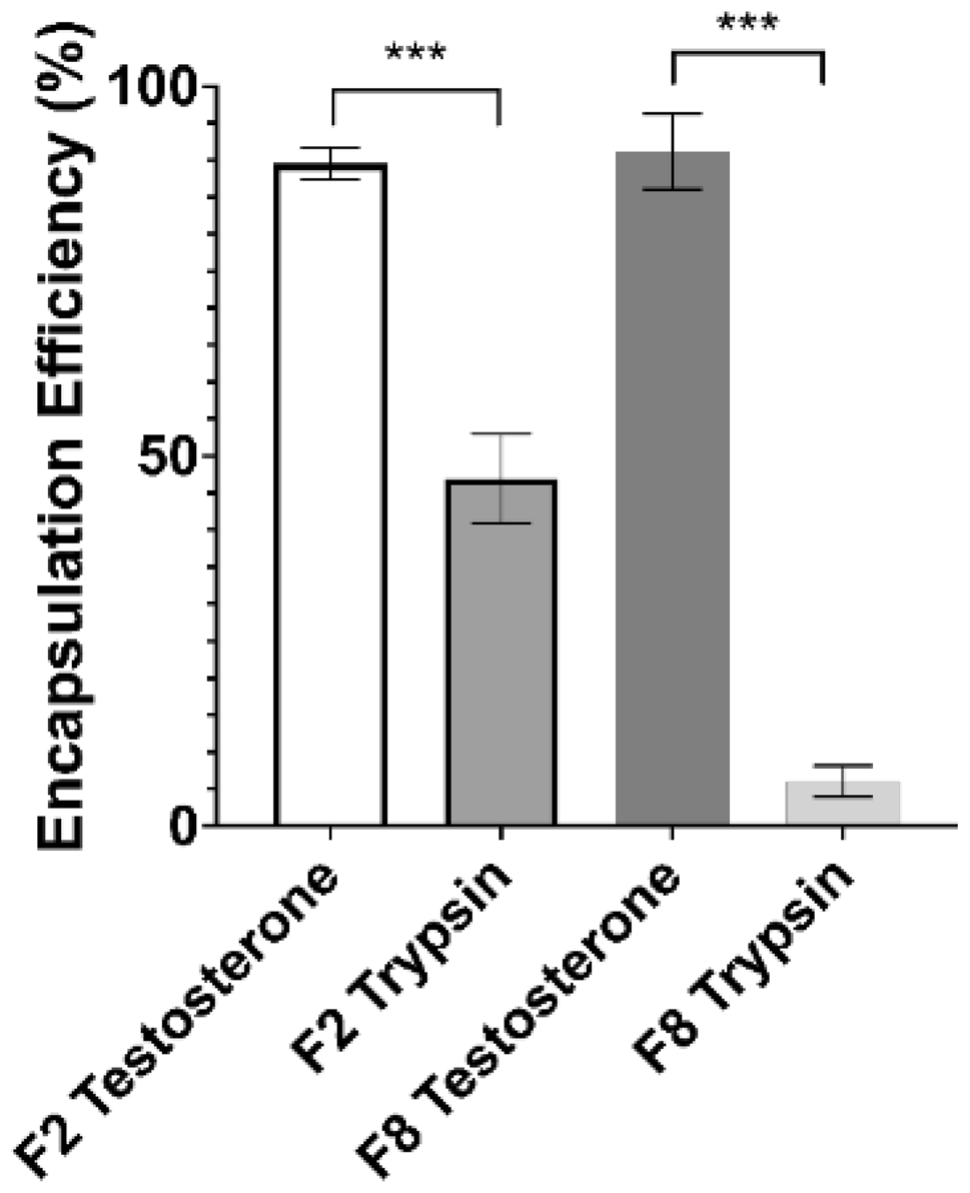
Encapsulation efficiency for F2 and F8 formulations encapsulating both TRP and TES

TES was used as a model small lipophilic drug which has been encapsulated previously within SLNs, although to the authors’ knowledge, not using MFs. As displayed in the EE data (Fig. 13), the EE of TES is extremely high for both chosen formulations. This has been slightly improved upon using MFs as compared to other methods such as emulsification/homogenisation [56, 57], although not to a dramatic degree. Due to the novelty of the process, the encapsulation of TRP within SLNs has not been attempted before so there are no direct comparisons that can be made; however, encapsulation of other biologics such as insulin within SLNs has been attempted and shows varying degrees of success, with the highest efficiency being circa 62% [58]. With a molecular weight of 24 kDa, TRP is a far larger biologic than has been previously attempted and an EE of 47% shows great promise for the MF method of SLN production. Due to the relatively high aqueous solubility of TRP caused mainly by Pin-II protease inhibitor hydrogen bonding and the prevalence of cysteine disulphide bonds throughout, the low EE obtained for F8 comes as no surprise, as only T80 provides a hospitable environment to encapsulate a hydrophilic material. TRP has hydrophobic regions in its tertiary structure at pH 7.4, owing to the presence of aspartic acid and tyrosine residues, so it is likely that a small amount of SLN association is due also to this factor. F2 has the capacity to provide a more prevalent hydrophilic region, due to the P68 surfactant layer, hence the higher EE possible with this formulation. For this reason, when considering EE alone, F2 would be deemed most suitable as a lead formulation. The EE for formulations for F1, F3, F5, and F7 are available to view in Online Resource 13, and a clear difference in encapsulation potentials for both TRP and TES can be seen via direct comparison. Most assayed formulations had a relatively high propensity for TES encapsulation, owing to the waxy nature of SLNs; however, the capacity to feasibly facilitate TRP encapsulation was only present in F1 and F2. This is suggestive evidence that the P68 surfactant layer indeed provides enhanced chemical conditions for TRP acceptance; however, only when coupled with a CP core material can it achieve relatively high TRP encapsulation. F5 also contains P68, but achieves an EE of just 10.5%, which is unsuitable for lead formulation selection. When equating to API concentrations in milligrams per millilitre, the concentrations achieved for EE for F2 are 22.25 ± 0.56 mg/ml and 0.47 ± 0.05 mg/ml respectively for TES and TRP, and for F8 are 22.80 ± 1.34 mg/ml and 0.06 ± 0.01 mg/ml for TES and TRP respectively.

**Fig. 14:**
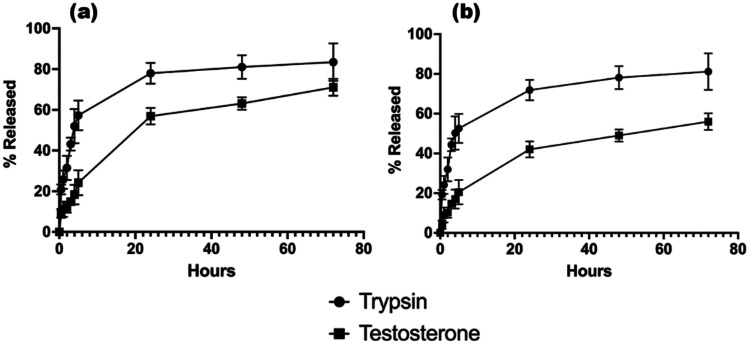
Drug release displayed as % total release from encapsulated active pharmaceutical ingredient for **a** F2 and **b** F8

The drug release demonstrated a sustained release with a noticeable difference between TRP and TES. As frequently mentioned in literature, release from SLNs is often slower than what may be displayed by liposomal formulations, due to the nature of the solid core [59]. This property is most apt for TES, due to its association within the core, and this also explains the slower release from the formulation, despite a far smaller size and diffusion potential. As displayed in Fig. 14, within the 72-h assayed period, TES formulations reached around 65% and 45% release of encapsulated API for F2 and F8 respectively, which would equate within the therapeutic range for TES therapy if external factors such as degradation kinetics and excretion were negated [60, 61]. This release equates to 14.5 mg/ml and 10.2 mg/ml for F2 and F8 respectively, not taking into account for clearance in vivo after the 72-h release period*.* Due to the low EE potential of F8, it is unlikely that this would be a plausible formulation for TRP, although as TRP represents a model drug, the data is not available to deem this a certainty. The TRP model suggests that the hydrophobicity of the lipid core of SLNs would make them appear to be an inadequate carrier for biological hydrophilic substances. In spite of this, SLNs proved to be a promising carrier for biologics due to the great protection they can provide from the rapid in vivo degradation. Each situation, however, should be carefully examined in order to maximise the EE of biologicals as lysozyme or insulin, taking into account the potential for using the biologic in its indissociated form to improve the EE into the lipid phase or through the choice of a different surfactant more suitable for the interaction with the hydrophilic molecule.

In fact, F2 displays a promising formulation for encapsulation of both hydrophilic and lipophilic APIs and in the case of TRP provides a controlled release with an initial burst profile as commonly seen from nanoformulations. TRP possesses a much more exaggerated burst release profile than the TES, owing to the partitioning as mentioned before.

### Process feasibility and scale-up

As can be seen by the results obtained, the quality of SLN produced via MFs is very high, although the matter of ease of production should be discussed. As explained in the methodology, the temperature required for formulation is 60 °C, to ensure components remain in solution and sufficient entropy is present. This temperature may be unsuitable for a number of biologic and peptide-based APIs, due to the high temperatures causing denaturation of secondary/tertiary structures. There are, however, a number of extremophile biologics, such as γ-lactamase [62] and various proteases/lipases [63], which are already used in such conditions, that could be translated to the method proposed in this manuscript.

The nature of MFs being a continuous process is very attractive for scale-up processes in an industrial setting, and with the added benefit of the decreasing price of MF systems, a parallel-run manufacturing line could be a possibility to allow for the production of a medicine at a wide scale. The risk of capillary blockage during the process is however a possibly; should the system fall below the required temperature, a knock-on effect of material precipitation could be caused. If this were to occur in an industrial situation, the delay caused by capillary clearance/replacement could be timely and inefficient. This is unlikely to occur in a GMP setting due to the number of backup protocols that would exist, but it is an important factor to consider for reproducing the data.

The production of SLNs via tradition methods is a lengthy process that requires large volumes of solvents in a batch process, at similar temperature conditions to what is being proposed by using MFs. The reduction of time needed to produce formulations with reduced solvent usage makes the MF process far more environmentally sustainable, which is an essential factor that is being considered by most pharmaceutical industries when concerning the production of medicines [28].

## Conclusion

The capacity to produce SLNs has been expanded using MFs using the method surmised in this manuscript. It is clear that the choice of SLN materials is important to cater for the desired API(s), owing to differing encapsulation potentials. In the case of the research performed in this manuscript, it appeared that F2, consisting of low-concentration CP and P68, provided the most promising lead formulation, although to bolster this claim, it would be effective to attempt the incorporation of other biologic molecules, as mentioned in the ‘Process feasibility and scale-up’ section. The novel method displayed represents a reproduceable and environmentally friendly method to encapsulate molecules that have previously been unfeasible to attempt and acts as a solid basis for further exploration.

### Supplementary Information

Below is the link to the electronic supplementary material.

Supplementary file1 (DOCX 2282 kb)

## Data Availability

The datasets generated during and/or analysed during the current study are available from the corresponding author on reasonable request.

## Abbreviations

MFs: Microfluidics
SLN: Solid lipid nanoparticle
API: Active pharmaceutical ingredient
LP: Liposome
NIO: Niosome
EE: Encapsulation efficiency
TFR: Total flow rate
FRR: Flow rate ratio
CP: Cetyl palmitate
P68: Pluronic 68
T80: Tween 80
DLS: Dynamic light scattering
DSC: Differential scanning calorimetry
FTIR: Fourier transform infrared spectroscopy
TRP: TRP
TES: Testosterone
LEC: Soybean lecithin
