# Supplementary material for: Microfluidic encapsulation of enzymes and steroids within solid lipid nanoparticles
Source: Drug Deliv Transl Res. 2023 Jul 28;14(1):266–79. doi: 10.1007/s13346-023-01398-5 (PMC10746583; doi:10.1007/s13346-023-01398-5)
Supplement: Supplementary file 1 — Supplementary file1 (DOCX 2282 kb) [file 13346_2023_1398_MOESM1_ESM.docx]

**Microfluidic Encapsulation of Enzymes and Steroids within Solid Lipid Nanoparticles**

Edward Weaver^1^, Federica Sommonte^1,2^, Andrew Hooker^3^, Nunzio Denora^2^, Shahid Uddin^3^, Dimitrios A. Lamprou^1,*^

^1^School of Pharmacy, Queen’s University Belfast, 97 Lisburn Road, Belfast, BT9 7BL, UK

^2^Department of Pharmacy - Pharmaceutical Sciences, University of Bari "Aldo Moro", 4 Orabona St., 70125, Bari, Italy

^3^Immunocore Ltd., 92 Park Dr, Milton, Abingdon, OX14 4RY, UK

*Correspondence to: D.Lamprou@qub.ac.uk (Dimitrios A. Lamprou)

**Supplementary Information**


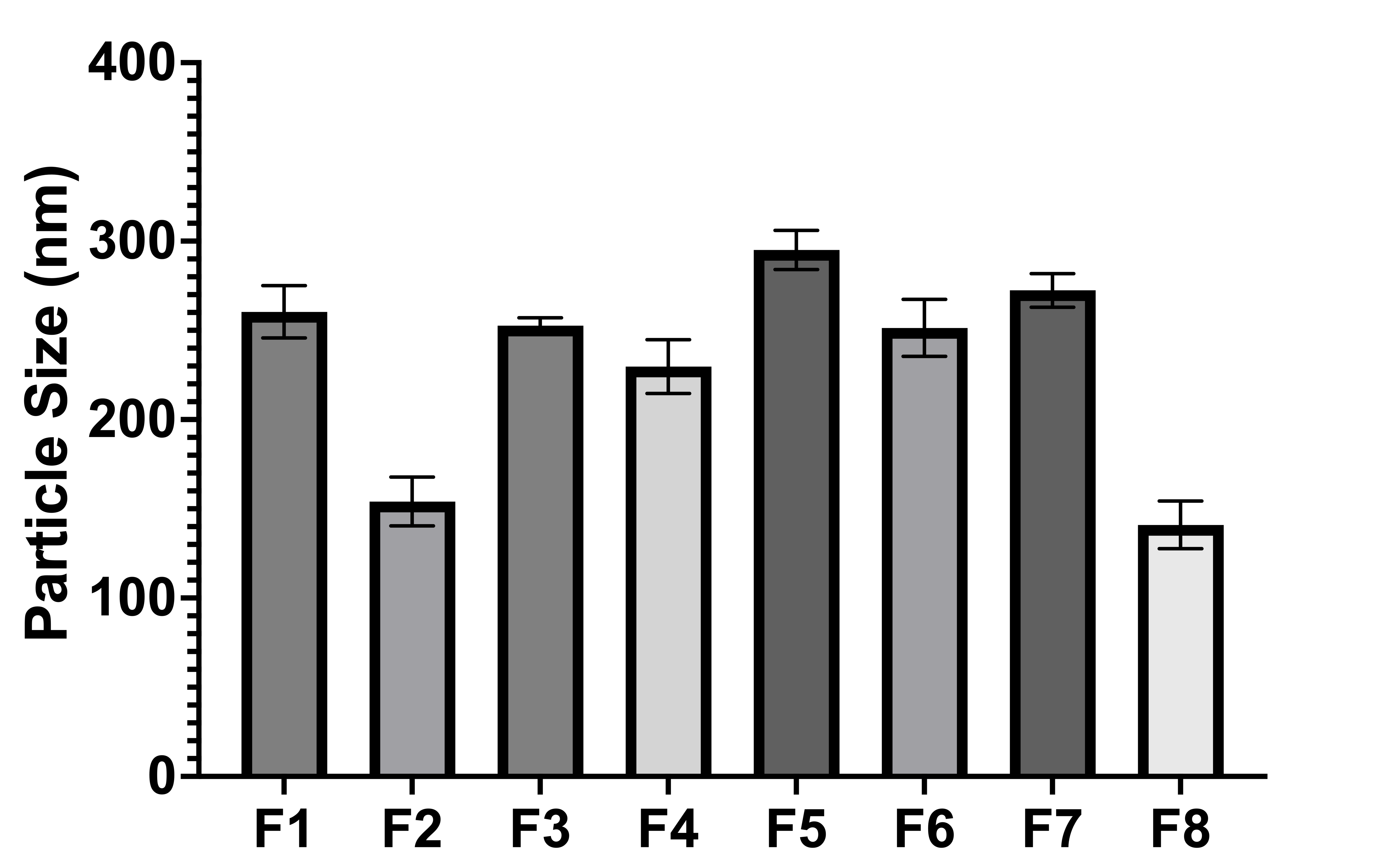


**Online Resource 1**. Particle size results obtained for empty SLNs – information on F1-F8 components can be found in table 1.


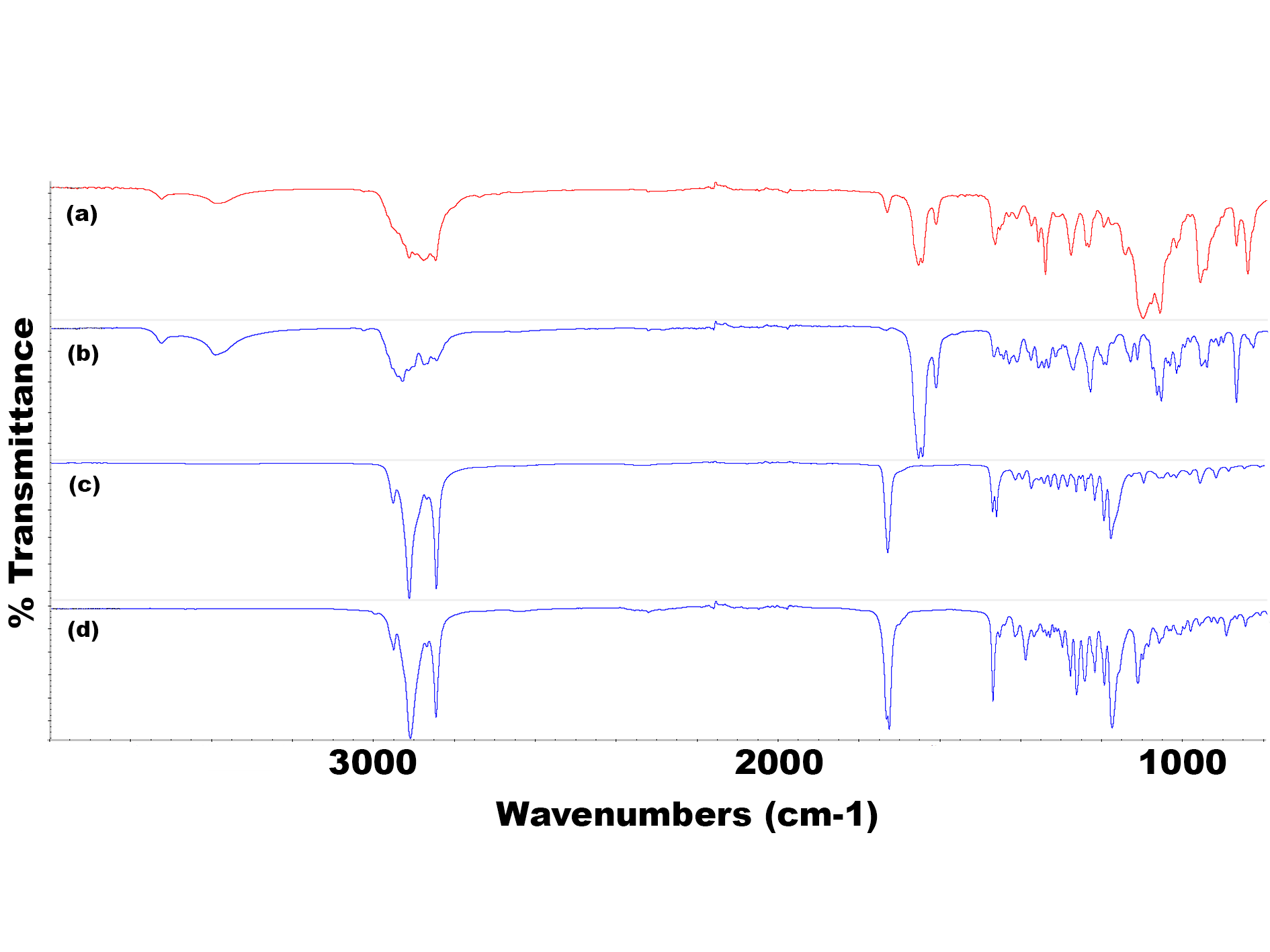


**Online Resource 2**. FTIR Spectra for all components of F2 TES Depicting (a) F2 TES-encapsulated SLNs (b) TES (c) CP and (d) P68.


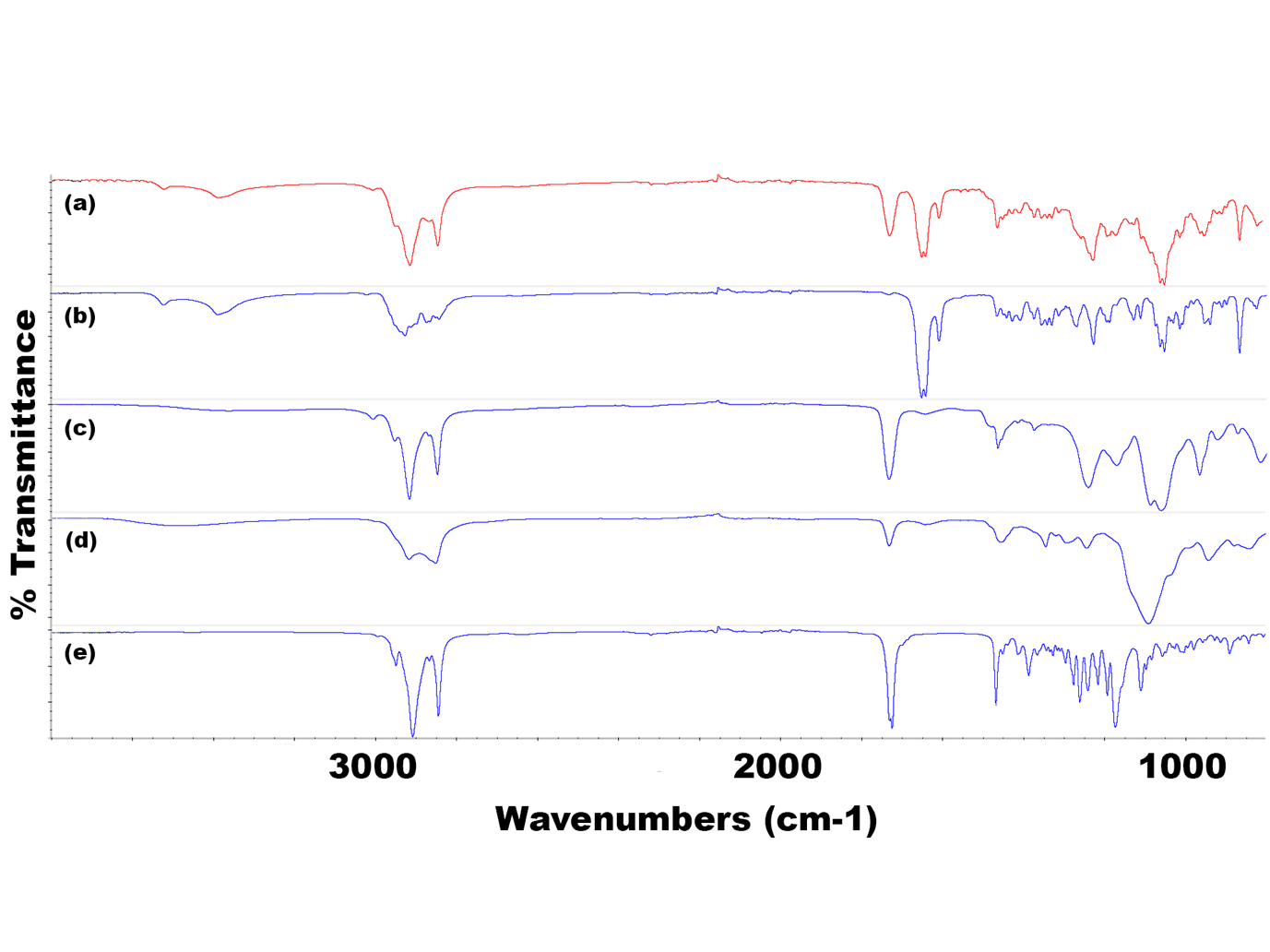


**Online Resource 3.** FTIR Spectra for all components of F8 TES Depicting (a) F8 TES-encapsulated SLNs (b) TES (c) LEC, (d) T80 and (e) Tri-P.


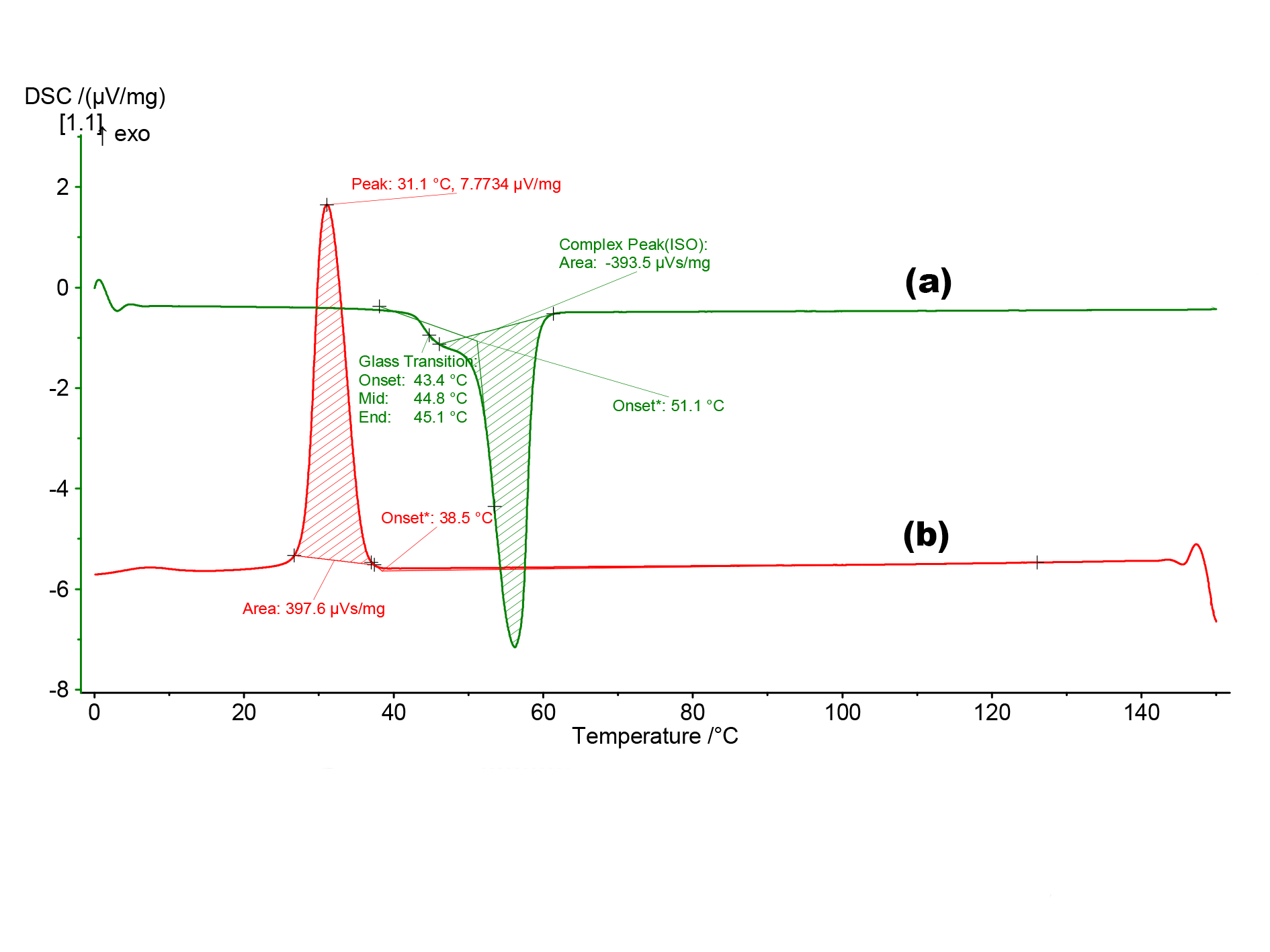


**Online Resource 4.** DSC Thermogram for Pluronic F68 representing (a) Heating curve and (b) Cooling curve.

|  | **F1** | | | |
| --- | --- | --- | --- | --- |
|  | 5°C | | | |
|  | Trypsin | | Testosterone | |
| Day | PDI | Zeta-Potential (mV) | PDI | Zeta-Potential (mV) |
| 0 | 0.234 | -15.26 | 0.222 | -18.58 |
| 7 | 0.214 | -14.29 | 0.216 | -17.44 |
| 14 | 0.241 | -10.22 | 0.218 | -17.22 |
| 21 | 0.353 | -11.42 | 0.251 | -18.46 |
| 28 | 0.344 | -9.85 | 0.246 | -15.93 |
|  | 37°C | | | |
| Day | PDI | Zeta-Potential (mV) | PDI | Zeta-Potential (mV) |
| 0 | 0.234 | -15.26 | 0.222 | -18.58 |
| 7 | 0.225 | -10.49 | 0.257 | -15.23 |
| 14 | 0.216 | -5.16 | 0.303 | -16.99 |
| 21 | 0.396 | -7.26 | 0.315 | -15.80 |
| 28 | -0.385 | -5.41 | 0.299 | -15.34 |

**Online Resource 5.** Stability study data for F1 for both TRP and TES. Data shows PDI and Zeta-potential over 5°C and 37°C over the course of the 28-day period.

|  | **F2** | | | |
| --- | --- | --- | --- | --- |
|  | 5°C | | | |
|  | Trypsin | | Testosterone | |
| Day | PDI | Zeta-Potential (mV) | PDI | Zeta-Potential (mV) |
| 0 | 0.195 | -16.46 | 0.204 | -20.16 |
| 7 | 0.193 | -14.22 | 0.201 | -21.86 |
| 14 | 0.197 | -14.95 | 0.186 | -19.28 |
| 21 | 0.193 | -14.90 | 0.183 | -16.46 |
| 28 | 0.204 | -12.10 | 0.191 | -17.31 |
|  | 37°C | | | |
| Day | PDI | Zeta-Potential (mV) | PDI | Zeta-Potential (mV) |
| 0 | 0.195 | -16.46 | 0.204 | -20.16 |
| 7 | 0.159 | -14.24 | 0.222 | -18.43 |
| 14 | 0.201 | -10.95 | 0.261 | -18.55 |
| 21 | 0.194 | -11.43 | 0.255 | -16.45 |
| 28 | 0.199 | -9.50 | 0.242 | -15.07 |

**Online Resource 6.** Stability study data for F2 for both TRP and TES. Data shows PDI and Zeta-potential over 5°C and 37°C over the course of the 28-day period.

|  | **F3** | | | |
| --- | --- | --- | --- | --- |
|  | 5°C | | | |
|  | Trypsin | | Testosterone | |
| Day | PDI | Zeta-Potential (mV) | PDI | Zeta-Potential (mV) |
| 0 | 0.196 | -15.44 | 0.206 | -18.42 |
| 7 | 0.209 | -9.45 | 0.210 | -14.63 |
| 14 | 0.355 | -14.07 | 0.280 | -14.88 |
| 21 |  |  | 0.291 | -15.02 |
| 28 |  |  |  |  |
|  | 37°C | | | |
| Day | PDI | Zeta-Potential (mV) | PDI | Zeta-Potential (mV) |
| 0 | 0.196 | -15.44 | 0.206 | -18.42 |
| 7 | 0.180 | -13.07 | 0.195 | -15.04 |
| 14 | 0.266 | -14.55 | 0.227 | -16.94 |
| 21 | 0.347 | -10.46 | 0.260 | -13.08 |
| 28 | 0.350 | -6.92 | 0.228 | -14.48 |

**Online Resource 7.** Stability study data for F3 for both TRP and TES. Data shows PDI and Zeta-potential over 5°C and 37°C over the course of the 28-day period. Any “blacked-out” data points represents the stage at which stability study was halted due to unsuitability of the formulation.

|  | **F4** | | | |
| --- | --- | --- | --- | --- |
|  | 5°C | | | |
|  | Trypsin | | Testosterone | |
| Day | PDI | Zeta-Potential (mV) | PDI | Zeta-Potential (mV) |
| 0 | 0.206 | -18.28 | 0.209 | -13.81 |
| 7 | 0.229 | -15.23 | 0.217 | -9.05 |
| 14 |  |  | 0.296 | -13.29 |
| 21 |  |  |  |  |
| 28 |  |  |  |  |
|  | 37°C | | | |
| Day | PDI | Zeta-Potential (mV) | PDI | Zeta-Potential (mV) |
| 0 | 0.206 | -18.28 | 0.209 | -13.81 |
| 7 | 0.194 | -10.43 | 0.226 | -14.02 |
| 14 | 0.259 | -6.04 | 0.207 | -15.03 |
| 21 | 0.304 | -12.53 | 0.261 | -13.57 |
| 28 | 0.323 | -9.44 | 0.289 | -13.06 |

**Online Resource 8.** Stability study data for F4 for both TRP and TES. Data shows PDI and Zeta-potential over 5°C and 37°C over the course of the 28-day period. Any “blacked-out” data points represents the stage at which stability study was halted due to unsuitability of the formulation.

|  | **F5** | | | |
| --- | --- | --- | --- | --- |
|  | 5°C | | | |
|  | Trypsin | | Testosterone | |
| Day | PDI | Zeta-Potential (mV) | PDI | Zeta-Potential (mV) |
| 0 | 0.183 | -22.51 | 0.201 | -24.70 |
| 7 | 0.195 | -23.48 | 0.219 | -20.52 |
| 14 | 0.248 | -20.02 | 0.242 | -16.31 |
| 21 | 0.285 | -18.45 | 0.240 | -17.89 |
| 28 | 0.274 | -16.00 | 0.249 | -16.25 |
|  | 37°C | | | |
| Day | PDI | Zeta-Potential (mV) | PDI | Zeta-Potential (mV) |
| 0 | 0.183 | -22.51 | 0.201 | -24.70 |
| 7 | 0.209 | -19.41 | 0.207 | -22.60 |
| 14 | 0.242 | -15.06 | 0.227 | -20.93 |
| 21 | 0.294 | -13.05 | 0.242 | -21.01 |
| 28 | 0.311 | -15.68 | 0.258 | -16.46 |

**Online Resource 9.** Stability study data for F5 for both TRP and TES. Data shows PDI and Zeta-potential over 5°C and 37°C over the course of the 28-day period.

|  | **F6** | | | |
| --- | --- | --- | --- | --- |
|  | 5°C | | | |
|  | Trypsin | | Testosterone | |
| Day | PDI | Zeta-Potential (mV) | PDI | Zeta-Potential (mV) |
| 0 | 0.189 | -12.62 | 0.177 | -11.30 |
| 7 | 0.208 | -10.95 | 0.216 | -11.28 |
| 14 | 0.298 | -7.02 | 0.307 | -9.41 |
| 21 |  |  |  |  |
| 28 |  |  |  |  |
|  | 37°C | | | |
| Day | PDI | Zeta-Potential (mV) | PDI | Zeta-Potential (mV) |
| 0 | 0.189 | -12.62 | 0.177 | -11.30 |
| 7 |  |  | 0.285 | -10.03 |
| 14 |  |  |  |  |
| 21 |  |  |  |  |
| 28 |  |  |  |  |

**Online Resource 10.** Stability study data for F6 for both TRP and TES. Data shows PDI and Zeta-potential over 5°C and 37°C over the course of the 28-day period. Any “blacked-out” data points represents the stage at which stability study was halted due to unsuitability of the formulation.

|  | **F7** | | | |
| --- | --- | --- | --- | --- |
|  | 5°C | | | |
|  | Trypsin | | Testosterone | |
| Day | PDI | Zeta-Potential (mV) | PDI | Zeta-Potential (mV) |
| 0 | 0.227 | -25.60 | 0.229 | -22.46 |
| 7 | 0.215 | -22.14 | 0.243 | -20.65 |
| 14 | 0.245 | -15.91 | 0.255 | -14.99 |
| 21 |  |  | 0.274 | -15.01 |
| 28 |  |  | 0.286 | -15.08 |
|  | 37°C | | | |
| Day | PDI | Zeta-Potential (mV) | PDI | Zeta-Potential (mV) |
| 0 | 0.227 | -25.60 | 0.229 | -22.46 |
| 7 | 0.229 | -18.41 | 0.246 | -18.76 |
| 14 | 0.246 | -15.83 | 0.253 | -15.48 |
| 21 |  |  | 0.288 | -16.94 |
| 28 |  |  |  |  |

**Online Resource 11.** Stability study data for F7 for both TRP and TES. Data shows PDI and Zeta-potential over 5°C and 37°C over the course of the 28-day period. Any “blacked-out” data points represents the stage at which stability study was halted due to unsuitability of the formulation.

|  | **F8** | | | |
| --- | --- | --- | --- | --- |
|  | 5°C | | | |
|  | Trypsin | | Testosterone | |
| Day | PDI | Zeta-Potential (mV) | PDI | Zeta-Potential (mV) |
| 0 | 0.166 | -23.57 | 0.170 | -25.44 |
| 7 | 0.181 | -22.10 | 0.184 | -22.07 |
| 14 | 0.209 | -24.68 | 0.197 | -19.46 |
| 21 | 0.183 | -19.39 | 0.193 | -16.92 |
| 28 | 0.194 | -19.92 | 0.202 | -18.73 |
|  | 37°C | | | |
| Day | PDI | Zeta-Potential (mV) | PDI | Zeta-Potential (mV) |
| 0 | 0.166 | -23.57 | 0.170 | -25.44 |
| 7 | 0.200 | -20.46 | 0.187 | -23.61 |
| 14 | 0.201 | -20.45 | 0.206 | -21.55 |
| 21 | 0.208 | -18.96 | 0.222 | -22.58 |
| 28 | 0.210 | -14.46 | 0.235 | -20.93 |

**Online Resource 12.** Stability study data for F8 for both TRP and TES. Data shows PDI and Zeta-potential over 5°C and 37°C over the course of the 28-day period.


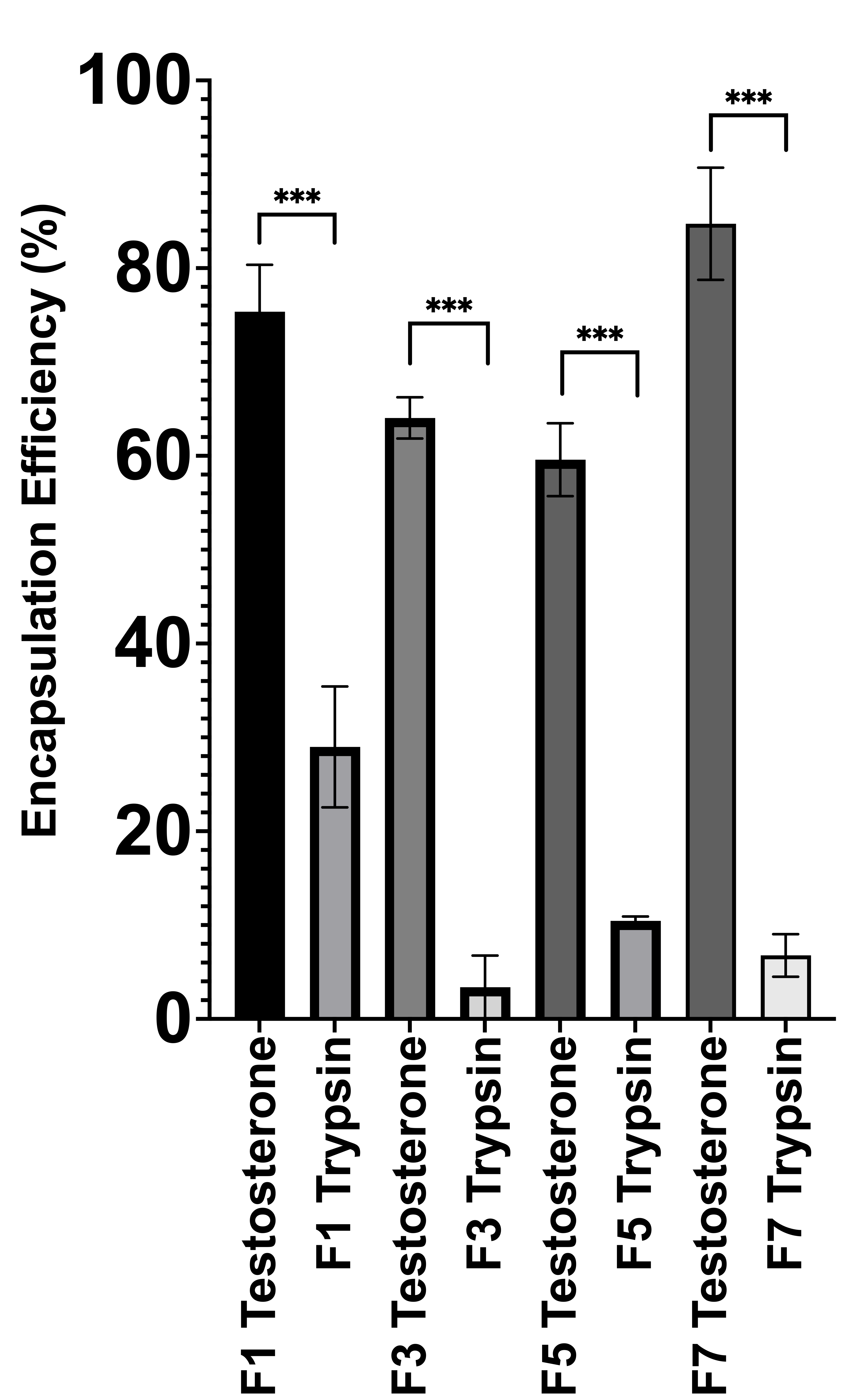


**Online Resource 13.** EE data displayed for both TRP and TES for formulations F1, F3, F5 and F7. EE for F6 and F4 omitted due to unfeasibility of formulation determined by stability data.
